# Anisotropic phonon dynamics in Dirac semimetal PtTe_2_ thin films enabled by helicity-dependent ultrafast light excitation

**DOI:** 10.1038/s41377-024-01540-z

**Published:** 2024-08-01

**Authors:** Ziyang Li, Yequan Chen, Anke Song, Jinzhong Zhang, Rong Zhang, Zongzhi Zhang, Xuefeng Wang

**Affiliations:** 1 Key Laboratory of Micro and Nano Photonic Structures (MOE), School of Information Science and Technology, Fudan University, Shanghai, China; 2 Jiangsu Provincial Key Laboratory of Advanced Photonic and Electronic Materials, State Key Laboratory of Spintronics Devices and Technologies, School of Electronic Science and Engineering, Collaborative Innovation Center of Advanced Microstructures, Nanjing University, Nanjing, China; 3 Department of Physics, School of Physics and Electronic Science, East China Normal University, Shanghai, China; 4 Department of Physics, Xiamen University, Xiamen, China

**Keywords:** Ultrafast photonics, Magneto-optics

## Abstract

Coherent phonons have aroused considerable attention in condensed matter physics owing to their extraordinary capacity of reflecting and controlling the physical properties of matter. However, the investigation on the interaction between coherent phonons and other microscopic particles on the ultrafast timescale within topological systems continues to be an active and unresolved area. Here, we show the energy transfer of coherent optical phonons (COP) in Dirac semimetal PtTe_2_ thin films using ultrafast optical pump-probe spectroscopy. Specifically, the helicity-dependent light-driven anisotropic COP signals disclose their direct connection with the light-excited anisotropic spin-polarized electrons via an angular momentum transfer. Furthermore, we observe the notable decreases in the COP oscillation frequency and the decay rate with increasing temperatures due to the anharmonic phonon-phonon scattering and electron-phonon scattering in the COP dissipation process, respectively. Our work paves the way for uncovering the coherent phonons in Dirac semimetals for the potential applications in optoelectronics and opto-spintronics.

## Introduction

In condensed matter physics, topological Dirac semimetals^1–4^ have attracted great interest due to their unique electronic band structures, which provide an ideal platform to investigate topological phase transition and the low-energy excitation of elementary particles^5,6^. PtTe_2_, a 2D layered van der Waals material in the transition metal dichalcogenide (TMD) family, possesses type-II Dirac fermions with a pair of strongly tilted and anisotropic Dirac cones^7^. The de Haas-van Alphen quantum oscillations further disclose an anisotropic Dirac band structure, which is accompanied with the low effective mass and the high carrier mobility^8,9^. In view of the high charge-to-spin conversion efficiency^10^, superior high conductivity^11,12^, excellent air stability^13^, dimensionality-mediated semimetal-semiconductor transition^14–16^, and broadband photoresponse from visible to terahertz range^15,17^, PtTe_2_ has become a promising candidate for spintronic^10^ and optoelectronic devices^15,17–20^. Very recently, the helicity-dependent terahertz emission has been observed in the inversion-symmetry-broken PtTe_2_ thin films^21^, further expanding the potential application range.

Coherent phonons represent the collective lattice vibrations that are in-phase, classified as coherent optical phonons (COP) and coherent acoustic phonons (CAP), which are recognized as effective tools for controlling fundamental physical properties and exploring the ultrafast dynamic processes of spins and carriers^22,23^. For example, through coherent phonons researchers have demonstrated the manipulation of macroscopic magnetic states in orthoferrite^24^, the ultrafast phase transitions from insulator to metal states^25^, and the enhancement of superconductivity^26^. Ultrafast optical pump-probe spectroscopy is a powerful tool for revealing the quasiparticle dynamics in the time domain. Through the time-resolved stimulated Raman scattering with picosecond laser pulses, Alfano and Shapiro firstly achieved the direct measurement of the COP lifetime in a calcite crystal^27^. Following this, they further observed the decay route of the methyl vibrations in ethanol^28^. Later, the exploration of lattice vibrations was expanded to a variety of conventional liquid and solid materials^29,30^. In recent years, the advancements of ultrafast laser techniques have facilitated the exploration of more COP-mediated interactions, enabling deeper understanding of complex physical phenomena in various quantum materials, such as topological insulators^22^, Weyl semimetals^23^, and Dirac semimetals^31^. For the Dirac semimetal PtTe_2_, most phonon studies only utilized the frequency-domain spontaneous Raman scattering^10,12,21^. Temporal behaviors of phonons, however, have rarely been reported in time-resolved measurements^32^. Therefore, it is urgent to explore the systematic temporal behaviors of coherent phonons to regulate the topological properties as well as optimize the performance of PtTe_2_-based optoelectronic devices.

In this work, the dynamics of coherent phonons in PtTe_2_ thin films is explored at various temperatures by ultrafast optical pump-probe technique. During the photoexcitation process, coherent phonons excited by the pump beam periodically modulate the local dielectric constant, which is monitored by the transient changes of the polarization of the probe beam. We detect the COP of the *E*_g_ vibrational mode and the longitudinal CAP excited by circularly polarized (CP) light. Interestingly, the COP exhibit helicity-dependent amplitudes and phases, as well as obvious anisotropy with respect to the polarization direction of the probe beam. Such an anisotropic behavior is ascribed to the coupling between laser-excited spin-polarized electrons and COP. Temperature-dependent measurements of lattice dynamics further show a clear redshift of the *E*_g_-phonon frequency and an unusual decrease in the vibrational decay rate with increasing temperatures. These findings benefit a deep understanding of phonon dynamics for a myriad of quantum materials.

## Results

### The basic structural properties of PtTe_2_ films

**Fig. 1:**
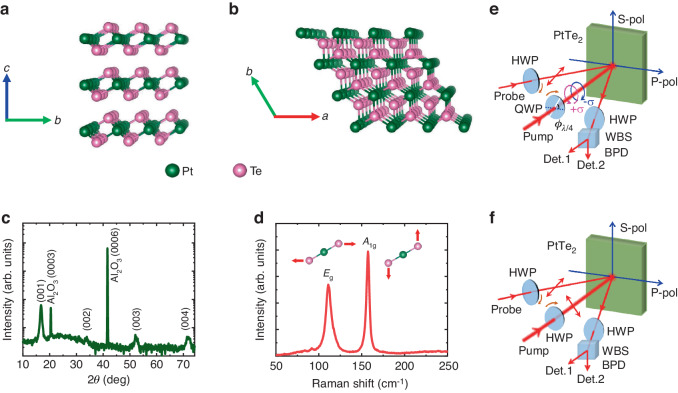
Crystal structure of PtTe_2_ and the optical pump-probe setup. **a**, **b** The side and top views of the PtTe_2_ crystal structure, respectively. Green and pink spheres represent the Pt and Te atoms, respectively. **c**, **d** The XRD pattern and Raman spectrum of PtTe_2_ films, respectively. **e**, **f** The schematic diagrams of the femtosecond laser pump-probe setup with elliptically polarized and linearly polarized pump beam excitation, respectively. The ellipticity angle *ϕ*_*λ*/4_ denotes the angle between P-polarization (P-pol) and fast axis of a quarter-wave plate. QWP quarter-wave plate. HWP half-wave plate. WBS Wollaston beam splitter. Det. 1 and Det. 2 a pair of channels of balanced photodetector (BPD). S-pol S-polarization

The high-quality PtTe_2_ thin films with a thickness of ~20 nm were grown on the sapphire substrates by pulsed laser deposition (PLD). Details of the sample growth process can be seen in “Materials and methods” section. The PtTe_2_ films possess a trigonal CdI_2_-type crystal structure with space group *P*$\bar{3}$*m*1 (NO. 164). It has a layered structure with the adjacent Te layers held together by van der Waals interaction in different monolayers and the Pt layer sandwiched between two Te layers in each monolayer, as schematically shown in the side and top views of Fig. 1a, b. Each Pt atom is surrounded by six Te atoms in a hexagonal periodic structure. Figure 1c displays a series of (00*n*) X-ray diffraction (XRD) peaks related to PtTe_2_ and sapphire, suggesting that these films are epitaxially grown along the [001] direction of the substrates. The Raman spectrum in Fig. 1d shows the characteristic peaks of *E*_g_ (~3.33 THz, 111 cm^–1^) and *A*_1g_ (~4.71 THz, 157 cm^–1^) vibration modes for 1*T*-PtTe_2_^33^. The *E*_g_ mode corresponds to in-plane relative motion of the bottom and top Te atoms in opposite directions, while *A*_1g_ is the out-of-plane vibration mode of the Te atoms.

### Ultrafast dynamics of PtTe_2_ films

The femtosecond laser pump-probe setups, with either elliptically polarized or linearly polarized pump beam excitation, are schematically displayed in Fig. 1e, f, respectively. An intense femtosecond pump beam is used to excite the lattice dynamics of PtTe_2_, which can be detected by the reflected probe beam passing through a balanced photodetector. The variation of polarization direction is acquired from the differential signals (Δ*I* = *I*_S_ − *I*_P_), which simultaneously reflect the transient changes of magneto-optical Kerr effect and anisotropic reflectivity (see “Materials and methods” section for details).

**Fig. 2:**
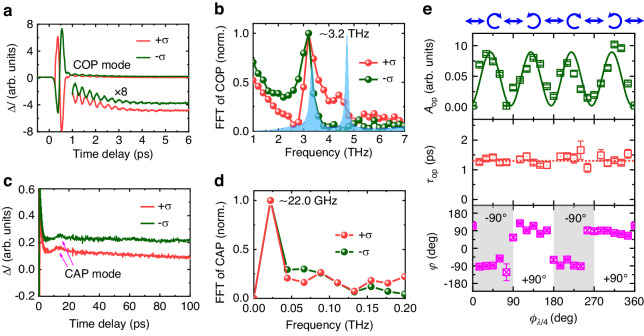
All-optical generation and detection of coherent phonons and helicity-dependent COP. **a** The photoinduced time-domain differential signals (Δ*I*) as a function of delay time. **b** The corresponding FFT spectra derived from (**a**). The blue shadow area in (**b**) shows the result from the Raman spectrum. **c**, **d** The transient signals for the longer time delay range and the corresponding FFT spectra. **e** The fitted parameters of *A*_op_, *τ*_op_, and *φ* versus *ϕ*_*λ*/4_. The solid green line is fitted with a sine function

As shown in Fig. 1e, we use the all-optical time-resolved pump-probe technique to measure the ultrafast dynamics of the PtTe_2_ films upon the right-handed (+*σ*, *ϕ*_*λ*/4_ = 45°) and left-handed (-*σ*, *ϕ*_*λ*/4_ = 135°) CP light excitation. The linearly-polarized (LP) probe beam is along the P-polarization (P-pol) direction. The ellipticity angle *ϕ*_*λ*/4_ of the pump beam is defined as the angle between P-pol and fast axis of a quarter-wave plate (QWP). Figure 2a shows the typical time-domain traces of the differential signals Δ*I* in the delay time range of 0–6 ps. In transient reflectivity or transmission spectroscopy, light excitation generally leads to an immediate rise/fall at nearly zero delay time due to photo-bleaching/photo-absorption, with the response time limited by the pulse duration of the femtosecond laser. However, an abrupt bipolar peak is observed when the delay time is within 1 ps with an inverted shape upon ±*σ* light excitation, which is attributed to optically spin-injection-induced Kerr rotation because the photoexcited hot electrons are transiently spin-polarized upon absorbing the angular momentum (±*ħ*) of ±*σ* photons^34^. From the subsequent spin relaxation process, we observe the spin depolarization within 1 ps and an attached high-frequency oscillation. Figure 2b exhibits the corresponding fast Fourier transform (FFT) frequency spectra, where a distinct terahertz mode with the central frequency ~3.2 THz can be recognized. The vibrational energy of ~13.23 meV matches *E*_g_ mode phonon in Raman spectrum, therefore, the oscillation mode is considered as the in-plane COP of *E*_g_ mode. Nevertheless, the component from out-of-plane COP of *A*_1g_ mode is negligible although the intensity of the *A*_1g_ mode is slightly greater than that of the *E*_g_ mode in Raman spectrum (Fig. 1d). Notably, the COP vibration undergoes a 180° phase shift as the pump light changes from +*σ* to –*σ*, which is closely linked to the spin-polarized electrons within the delay time of 1 ps.

Figure 2c shows the transient Δ*I* signal within a longer delay time range of 100 ps. A pair of valley and peak (marked with the purple arrows) can be identified during 6–13 ps, which corresponds to the lattice vibration of low-frequency longitudinal CAP with ~22 GHz (Fig. 2d). The oscillation of CAP signals exhibits only one full cycle, which is ascribed to the fact that the laser penetration length is comparable to the thickness of the sample (see Supplementary Note 1). Different from the opposite vibrational phase of COP, the CAP vibration remains in phase for the pump light with ±*σ* helicity, which is consistent with the origin of the laser heating effect. After subtracting the CAP and COP oscillation signals, the background dynamic process with time delay longer than 1 ps is well-fitted by a biexponential decay function, turning out two carrier lifetimes of *τ*_1_ ≈ 1 ps and *τ*_2_ > 100 ps. The transient *τ*_1_ is due to the hot-carrier cooling by electron-phonon scattering and the long-lived *τ*_2_ denotes the phonon-assisted electron-hole recombination^23^.

### Helicity-dependent phonons in PtTe_2_ films

To definitively determine the relation between optically spin injection and the COP feature, the time-domain traces are measured for pump pulses with variable elliptical polarization by rotating a QWP (Fig. 1e), namely changing angle *ϕ*_*λ*/4_ and fixing the LP probe beam at P-pol direction. The curves are fitted by the following equation^35^:

1 $$\begin{array}{ll}\Delta I(t)=&{D}_{0}+{A}_{{\rm{e}}-{\rm{ph}}}\exp \left(-\frac{t-{t}_{0}}{{\tau }_{{\rm{e}}-{\rm{ph}}}}\right)\\&+\,{A}_{{\rm{op}}}\exp \left(-\frac{t-{t}_{0}}{{\tau }_{{\rm{op}}}}\right)\sin [2\pi f(t-{t}_{0})+\varphi ]\end{array}$$

where *D*_0_ is a fitting constant related to the background signal, the second term represents the non-oscillatory signal resulting from the relaxation process of photoexcited hot carriers via electron-phonon scattering, which is independent of *ϕ*_*λ*/4_, and the last term is the oscillatory COP component. *A*_op_, *τ*_op_, *f*, and *φ* in the last term are the vibration amplitude, lifetime, frequency, and initial phase, respectively. The fitted constant value of *τ*_e-ph_ is about 1.2 ps, in good agreement with the reported electron-acoustic phonon scattering time^22^. The fitted *f* of COP is ~3.17 THz, coincident with the FFT result (Fig. 2b). Figure 2e shows the fitted *τ*_op_, *A*_op_, and *φ* as a function of *ϕ*_*λ*/4_. Specifically, the value of *τ*_op_ keeps almost unchanged (~1.3 ps), indicating that the interaction of photon, electron, and phonon just modulates the generation of COP at the very beginning upon laser excitation. While the following relaxation process of COP is mainly influenced by the intrinsic phonon-phonon and electron-phonon scattering of PtTe_2_, as discussed below. The helicity-dependent *A*_op_ shows a definite sine-function fitting, indicating only the helical light can excite the visible lattice vibration. The COP phase strictly relies on the laser helicity. As such, the pump light with opposite helicity drives the lattice motion in the opposite direction, indicating the connection between optically spin injection and the lattice dynamics of the COP component.

In general, there are two mechanisms responsible for the production of COP by ultrafast laser pulses, namely the impulsive stimulated Raman scattering (ISRS) in transparent materials^36^ and the displacive excitation of coherent phonon (DECP) in opaque materials^37^. ISRS enables the excitation of all Raman-active phonon modes with different symmetries, whereas DECP is limited to the fully symmetric modes that are displaced without varying the crystal symmetry. By examining the ultrafast absorption spectra^32^, a fully symmetric *A*_1g_ mode COP with a vibration frequency of ~4.68 THz is observed in the PtTe_2_ films due to the DECP mechanism. In other TMD materials such as WSe_2_^38^, TiTe_2_^39^, and NiTe_2_^31^, the displacive *A*_1g_-COP have been also detected by the optical pump-probe technique. Notably, the *E*_g_-COP are also observable in our PtTe_2_ films, which is usually absent in other TMD materials. Considering that the displacement of *E*_g_ vibration lowers the crystal symmetry, the *E*_g_-COP in the literature is commonly ascribed to the ISRS excitation^40^.

**Fig. 3:**
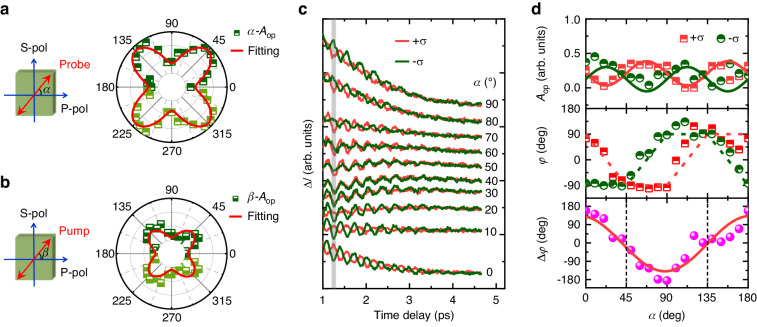
LP and CP laser-induced anisotropic phonon dynamics. **a**, **b** The extracted COP amplitudes of *E*_g_ mode versus angle *α* and *β* for LP excitation, respectively. The angle *α* (*β*) is defined as the angle between the linear polarization direction of probe (pump) beam and P-pol direction. The red solid curves represent the best fitted lines via the TSRS model. **c** The time-domain traces at various probe angles for CP excitation. The red (green) lines correspond to the pump excitation with +*σ* (–*σ*) helicity light. The curves are vertically shifted for clarity. The vertical grey line indicates the change in phase difference (Δ*φ*) between +*σ* and –*σ* excitation. **d** The fitted *A*_op_, *φ* and Δ*φ* as a function of *α*. The solid curves represent the fitted lines according to a sine function

The COP excitation by ISRS should obey the theory of transient stimulated Raman scattering (TSRS). In the TSRS model, the amplitude of coherent atomic motion is associated with the Raman tensor and the electric field directions of the LP pump and probe light^41^. However, the amplitude of purely displacive COP should be proportional to the density of photoexcitation electrons dependent on the optical absorption. To clarify this phenomenon, the time-resolved curves were measured after LP pump beam excitation. The polarization angle (*α*) of the incident probe beam varies from 0° to 180° by rotating a half-wave plate (HWP), while the LP pump beam is fixed along the P-pol direction (*β* = 0°) (Fig. 1f). The phonon vibrations become notably stronger when *α* approaches 45° and 135°, as shown in Fig. S1, and it is observed that the vibration phase is opposite between the two angular ranges of *α* = 0–90° and 90–180°. Figure 3a summarizes the values of *A*_op_ of *E*_g_-COP as a function of *α*, derived from the FFT spectra. Figure 3b shows the *β*-dependent *A*_op_, in which the polarization angle of the LP pump beam is varied (*β* = 0–180°) with the unchanged P-pol probe beam (*α* = 0°). According to the TSRS model, we theoretically deduce the formula of the vibration intensity of both the *E*_g_ and *A*_1g_ phonon modes (see Supplementary Note 2). Considering that the doubly degenerate *E*_g_ mode owns two second-order Raman tensors of ***R***(*E*_g_)_1_ and ***R***(*E*_g_)_2_, the calculated vibration intensity can be expressed as *S*_(*E*g)1_∝|2*c*^2^sin(2*α*)cos(2*β*)| and *S*_(*E*g)2_∝|2*c*^2^cos(2*α*)sin(2*β*)|, respectively. For the case of *β* = 0°, the *S*_(*E*g)2_ is negligible while *S*_(*E*g)1_ shows a fourfold symmetry with respect to *α*. This well accounts for the observed experimental results in Fig. 3a. Meanwhile, a *β*-dependent fourfold symmetry is also extracted from *S*_(*E*g)2_ at *α* = 0° (Fig. 3b). Therefore, the TSRS model can well explain the observed anisotropy of *A*_op_, confirming the ISRS mechanism in the LP excitation of *E*_g_-COP. However, the maximum *A*_op_ in Fig. 3b is significantly smaller than that in Fig. 3a, indicating that the contributions of two degenerate *E*_g_ modes are inequivalent. Moreover, the mode of ***R***(*E*_g_)_1_ possesses a more remarkable vibration than that of ***R***(*E*_g_)_2_, which has been demonstrated by the angle-resolved Raman scattering experiments (see Supplementary Notes 3 and 4).

The further investigation is implemented on the anisotropic lattice dynamics following ±σ CP excitation, as shown in Fig. 1e. Figure 3c exhibits a series of transient curve pairs measured with the different probe angle *α* after pumping with ±σ CP light. By fitting the curves using Eq. (1), the *A*_op_ and *φ* values as a function of *α* are obtained (Fig. 3d). The *A*_op_ exhibits a fourfold symmetry and has opposite anisotropy for ±σ pumping. Interestingly, the *φ* value displays a unique variation behavior as a function of *α*. As *α* increases from 0° to 45° (or from 90° to 135°), the oscillation phase *φ* of + *σ* excitation experiences a 180° shift, while that of –*σ* excitation remains almost unchanged. In contrast, when *α* increases from 45° to 90° (or from 135° to 180°), *φ* of –*σ* excitation also experiences a 180° shift. The phase difference (Δ*φ*) between the ±*σ* excitation is also summarized in Fig. 3d, which obeys a relation of Δ*φ* = 180°·sin(2*α* + 90°). It should be noted that similar anisotropy is obtained when the PtTe_2_ films are rotated by 45°, indicating that the anisotropy is attributed to the optical manipulation rather than PtTe_2_ crystal orientation.

**Fig. 4:**
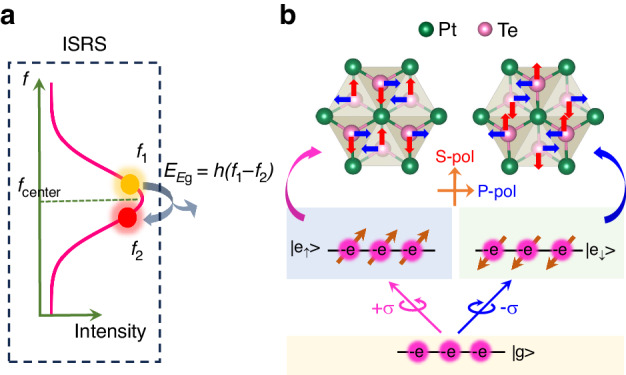
The physical picture of the anisotropic COP excited by CP light. **a** The illustration of COP generation by the ISRS mechanism. **b** Excitation of spin-polarized electrons, where |g> is the ground state and |e_↑_> (and |e_↓_>) is the spin-dependent excited state. The middle panel displays *E*_g_-mode oscillations excited by oppositely spin-polarized electrons, in which the red and blue arrows represent the vibrations of Te atoms in the S-pol direction and in the P-pol direction, respectively

The physical picture of COP generation via ISRS is schematically shown in Fig. 4a. During the ultrafast femtosecond laser pulse excitation, a photon of frequency *f*_1_ could be converted into another photon of the lower frequency *f*_2_, while releases an *E*_g_ mode phonon, satisfying the energy conservation^36^. ISRS can well explain the anisotropic behavior of LP light-driven COP, but cannot account for the exotic *α*-dependent *A*_op_ and Δ*φ* upon CP light excitation through the direct photon-phonon interaction. As discussed above, the COP signals via CP excitation are due to the optical spin injection, i.e., a DECP mechanism that the photoinduced spin-polarized electrons dominate the generation of COP. Here, two possible processes are proposed. First, after the helical light excitation, electrons with the opposite spin orientations at the ground state |g> are excited to a high-energy state with different orbitals (Fig. 4b) according to the spin selection rule^42^. The change of orbital electron distribution disturbs the equilibrium position of the atoms in the crystal, which may have an enhanced or suppressed effect on the *E*_g_-phonons in different vibrational directions. Second, since spin depolarization of the excited electrons experiences within 1 ps, the strong spin-lattice scattering can also modulate the observed COP vibrational behavior^43,44^, where the anisotropic distribution of electron spin may lead to the anisotropic COP behavior.

### Temperature-dependent phonons in PtTe_2_ films

**Fig. 5:**
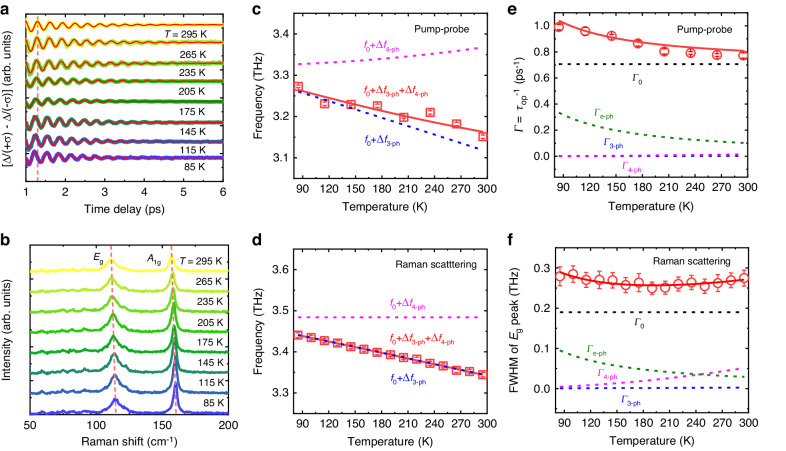
Temperature-dependent *E*_g_-mode optical phonons. **a**, **b** The time-domain traces of [Δ*I*(+*σ*) − Δ*I*(-*σ*)] and the frequency-domain Raman spectra measured at various temperatures, respectively. **c**, **d** The temperature-dependent frequency for *E*_g_-mode phonons derived from (**a**) and (**b**), respectively. **e**, **f** The inverse of COP lifetime (*τ*_op_^–1^) and the FWHM of *E*_g_-mode Raman peak as a function of temperature. The red solid lines are the best fitting using Eqs. (2–4) and Eqs. (5–7), respectively. The blue, purple, and green dashed lines show the corresponding contributions from three-phonon (3-ph), four-phonon (4-ph), and electron-phonon (e-ph) coupling, respectively

The temperature-dependent time-domain curves are shown in Fig. 5a to reveal the underlying dissipative mechanism of COP. The difference in transient signals between Δ*I* (+*σ*) and Δ*I* (-*σ*) rules out the background signals from hot carriers. It is also compared with the behavior of thermal phonons in frequency-domain spontaneous Raman scattering spectra, as displayed Fig. 5b, where the phonon frequency and the full width at half maximum (FWHM) of Raman shift peak are extracted by Lorentz fitting. By performing theoretical two-temperature model calculations in pump-probe technique and measuring the Stokes and anti-Stokes scattering in Raman spectroscopy, we demonstrate that the increase in local temperature due to laser heating is similar and minimal in both methods (see Supplementary Note 5). Therefore, its impact on the temperature-dependent experiments can be deemed negligible. Figure 5c, d depicts the temperature-dependent frequency of *E*_g_ mode by pump-probe technique and Raman spectroscopy, respectively. As temperature increases from 85 to 295 K, both curves exhibit similar linear trends, with a redshift value of ~0.11 (0.10) THz for pump-probe (Raman scattering) spectra. The frequency obtained by pump-probe method is 0.20 THz lower than that of Raman scattering, which is mainly due to the different mechanisms of the two measurement methods. In the intensive ultrafast pump-probe experiment, the absorption of femtosecond laser energy gives rise to a strong enhancement in electron temperature, leading to electron redistribution at the Fermi energy within several picoseconds. The laser-induced local increase in electron or hole density alters the interatomic interactions, resulting in large internal pressure^45^, which reduces the elastic constant and consequently softens the phonon mode. The relationship between phonon softening and photoexcited carrier density is verified by the pump-fluence-dependent experiment shown in Supplementary Note 6.

Usually, the temperature (*T*)-dependent frequency of optical phonons can be expressed by^46^

2 $$f(T)={f}_{0}+\Delta {f}_{{\rm{th}}}(T)+\Delta {f}_{{\rm{ph}}-{\rm{ph}}}(T)$$

where *f*_0_ accounts for the intrinsic phonon frequency at 0 K, Δ*f*_th_ corresponds to the frequency shift caused by thermal expansion of lattice parameters, and Δ*f*_ph-ph_ is the frequency shift associated with anharmonic phonon-phonon interaction. The contribution of thermal expansion is described by^47^

3 $$\Delta {f}_{{\rm{th}}}(T)={f}_{0}\left\{\exp [-m\gamma {\int }_{\!0}^{T}a(T^{\prime} )dT^{\prime} ]-1\right\}$$

where *m* denotes the degeneracy of the *E*_g_ mode (*m* = 2), *γ* is the Grüneisen parameter of the optical Raman mode, and *a*(*T*) represents the temperature*-*dependent linear lattice thermal expansion coefficient. It has been reported that the surface lattice constant of 1*T*-PtTe_2_ remains unchanged in the 90–580 K^48^, suggesting *a*(*T*) is close to zero and the lattice thermal expansion can be negligible. As a result, only the redshift of Δ*f*_ph-ph_ due to phonon-phonon anharmonicity is taken into account, which can be written as^46^

4 $$\Delta {f}_{{\rm{ph}}-{\rm{ph}}}(T)=A\,\left[1+\frac{2}{{e}^{x}-1}\right]+B\,\left[1+\frac{3}{{e}^{y}-1}+\frac{3}{{({e}^{y}-1)}^{2}}\right]$$

The first and second terms represent the three-phonon and four-phonon scattering processes, respectively. *A* and *B* are the corresponding fitting parameters. *x* = *hf*_0_/2*k*_B_*T* and *y* = *hf*_0_/3*k*_B_*T*, where *h* is the Planck constant and *k*_B_ is the Boltzmann constant. The consistence between the fitting results and the experimental data (Fig. 5c, d) indicates that the anharmonic phonon-phonon interaction is responsible for the energy change of the *E*_g_ phonons. With the temperature increasing, the four-phonon scattering contribution to frequency (Δ*f*_4-ph_) either increases (pump-probe method) or keeps almost constant (Raman spectra), indicating that the observed redshift is dominated by three-phonon scattering process.

Figure 5e shows the decay rate (*Γ* = 1/*τ*_op_^–1^) as a function of temperature of the *E*_g_-COP derived from Fig. 5a. The gradually decreasing *Γ* with increasing temperature cannot be simply demonstrated by the anharmonic phonon-phonon scattering model^31^. Another important optical phonon dissipation channel, namely electron-phonon coupling, should be considered, where the decoherence process of phonons is triggered by creating an electron-hole pair^49^. The temperature-dependent decay rate is written as follows^49^,

5 $$\varGamma (T)={\varGamma }_{0}+{\varGamma }_{{\rm{ph}}-{\rm{ph}}}(T)+{\varGamma }_{{\rm{e}}-{\rm{ph}}}(T)$$

where *Γ*_0_ is the intrinsic decay rate at 0 K, *Γ*_ph-ph_ and *Γ*_e-ph_ are the decay rate by anharmonic phonon-phonon and electron-phonon scattering, respectively. The *Γ*_ph-ph_ term is provided by^46^

6 $${\varGamma }_{{\rm{ph}}-{\rm{ph}}}(T)=C\left[1+\frac{2}{{e}^{x}-1}\right]+D\left[1+\frac{3}{{e}^{y}-1}+\frac{3}{{({e}^{y}-1)}^{2}}\right]$$

where the fitting parameters *C* and *D* represent the three-phonon and four-phonon scattering, respectively. Meanwhile, the temperature-dependent *Γ*_e-ph_ can be described as^49^

7 $${\varGamma }_{{\rm{e}}-{\rm{ph}}}(T)={\varGamma }_{{\rm{e}}-{\rm{ph}},0}\,\left[\frac{1}{{e}^{-x}+1}-\frac{1}{{e}^{x}+1}\right]$$

where *Γ*_e-ph,0_ is the residual decay rate from electron-phonon interaction at 0 K. The decay rate *Γ*_e-ph_ is proportional to the difference between the number of occupied states of carriers above and below the Fermi level in the energy range of *hf*_0_. According to the Fermi-Dirac distribution, electrons above the Fermi surface and holes below the Fermi surface could inevitably increase with increasing temperature due to the enhanced thermal excitation^49,50^. As a result, the electron-hole pairs excited by optical phonons would be suppressed, giving rise to the observed decreasing tendency of *Γ*_e-ph_. The corresponding fitting based on Eqs. (5–7) is shown in Fig. 5e. The different contributions of *Γ*_0_, *Γ*_e-ph_, *Γ*_3-ph_, and *Γ*_4-ph_ are illustrated by dashed lines, in which the value of electron-phonon coupling is much larger than that of phonon-phonon coupling. In addition, only *Γ*_e-ph_ drops with increasing temperature, similar to the total decay rate *Γ*, suggesting that the electron-phonon coupling is dominant. It should be noted that at the higher temperature, the increase rate of *Γ*_ph-ph_ will exceed the decrease rate of *Γ*_e-ph_, leading to a dominant rising behavior of the total decay rate *Γ*.

The FWHM of the *E*_g_ mode in Raman peak, i.e., the thermal phonon decay rate of *Γ*^46^, is shown in Fig. 5f. It is worth mentioning that the FWHM exhibits a definite nonmonotonic variation behavior as a function of temperature, with a minimum occurring at 210 K. This behavior is very similar to that of the G mode in graphite^51^ and *E*_g_ mode in PdTe_2_^52^, but completely differs from that of *A*_1g_ mode, as described in Supplementary Note 7. Obviously, the electron-phonon coupling dominates the decreasing process below 210 K, while the anharmonic phonon-phonon coupling turns to play a primary role above 210 K due to the enhanced contribution of four-phonon scattering.

According to the fitting parameters summarized in Supplementary Table S1 in Supplementary Note 8 and the correlation of *Γ*_e-ph,0_ = *λ*_e-ph_*f*_0_/4^49,51^, the dimensionless electron-phonon coupling strength coefficient *λ*_e-ph_ can be extracted. The *λ*_e-ph_ is 0.92 from the pump-probe technique and 0.24 from Raman scattering measurements, respectively, largely consistent with the theoretical calculations (0.35)^53^ and the previous experimental observation (0.38–0.42)^48^. These results obtained from time- and frequency-domain measurements suggest a similar dissipation mechanism of the *E*_g_-COP and thermal phonons in PtTe_2_ films. Furthermore, these results further verify that 1*T*-PtTe_2_ is a strong electron-phonon coupling material.

## Discussion

In conclusion, helicity-dependent excitation of *E*_g_-COP has been observed in Dirac semimetal PtTe_2_ thin films by using all optical pump-probe spectroscopy, showing the anisotropic oscillation amplitude and phase with respect to the probe light polarization. Unlike the LP light-driven COP that follows the ISRS mechanism, spin-polarized hot electrons dominate the COP vibration excited by ±*σ* light. In addition, it is revealed that the anharmonic phonon-phonon interaction and electron-phonon scattering determine the temperature-dependent frequency shift and dissipation process of COP, respectively. The electron-phonon coupling strength coefficient of *E*_g_ mode is obtained from the time-domain pump-probe and frequency-domain Raman spectroscopy measurements, respectively. This work provides an in-depth understanding of the ultrafast phonon dynamics in topological Dirac semimetals for the potential applications in optoelectronic and opto-spintronic devices.

## Materials and methods

### The growth of PtTe_2_ thin films

The 20-nm-thick PtTe_2_ thin films were grown on the sapphire (Al_2_O_3_) substrates (5 × 5 mm^2^) by the PLD technique designed for the epitaxial telluride films^54^. The target was prepared by heating the mixed Pt (99.99%) and Te (99.99%) powders with a stoichiometric ratio of 1:2.5 at 450 °C for four days. The basic pressure of the PLD vacuum chamber was maintained at 3 × 10^−5^ Pa. The PtTe_2_ films were deposited onto the substrates at 500 °C with a speed of ~1 nm min^–1^ using a 248 nm KrF excimer laser beam (fluence of 1 J cm^–2^ and repetition rate of 2 Hz).

### Time-resolved transient measurements

The optical pump-probe technique was used to measure the ultrafast dynamics. A pulsed Ti:sapphire laser (with a central wavelength of 800 nm, a pulse duration of 150 fs, and a repetition rate of 1 kHz) was split into an intensive pump beam (laser fluence of 400 μJ cm^−2^) under nearly normal incidence and a time-delayed weak probe beam at ~20° incidence with respect to the film normal direction. They were focused onto the same spot with a diameter of 0.8 and 0.2 mm, respectively^55^. Circularly-polarized, elliptically-polarized, and linearly-polarized pump pulses were utilized to excite the dynamic behaviors by rotating a QWP or HWP. The angle *ϕ*_*λ*/4_ of the pump beam is defined as the angle between P-pol and fast axis of a QWP. The incident probe beam was kept LP, with its polarization direction modulated by a HWP. The *α* (*β*) is defined as the angle between the LP direction of probe (pump) beam and P-pol direction. The reflected probe pulses entered a balanced photodetector after passing through a HWP and a Wollaston beam splitter. The differential signals (Δ*I* = *I*_S_ − *I*_P_) from the balanced photodetector were acquired with a lock-in amplifier. The setup can simultaneously detect the magneto-optical Kerr effect and anisotropic reflectivity.

### Raman scattering experiment

The Raman spectroscopic measurements were carried out on a micro-Raman spectrometer (LabRAM HR 800 UV) with a spectral resolution of 0.5 cm^−1^, as shown in Fig. S2a. A laser beam with a wavelength of 532 nm and a power of 1.0 mW (25% neutral density filter) was focused onto a spot with a diameter of ~1 μm through a 100× microscope objective (N. A. = 0.9). A multichannel air-cooled CCD (–70 °C) with 1024 × 256 pixels in the spectral range of 200–1050 nm was used to detect Raman scattering signals dispersed on a 1800 grooves/mm grating. Linkam THMSE 600 heating/cooling stage was utilized at a varying temperature rate of 10 K min^–1^ for the temperature-dependent Raman spectra at 85–295 K. In order to further check the anisotropy of the excited phonons in PtTe_2_, angle-resolved polarized Raman spectra were recorded in a backscattering geometry under the *θ*H and *θ*V scattering configuration. As shown in Fig. S2b and S2c, the film surface was lying the *x*-*y* plane and the incident laser was along the *z-*axis. *θ* is the angle between the direction of the incident LP light and the *y*-axis. The *θ*H configuration corresponded to the analyzer along the *x-*axis, while the *θ*V configuration was set with the analyzer parallel to the *y*-axis^56^. During the measurement, the sample was kept stationary and the polarization angle *θ* of the incident LP light was changed by rotating a HWP, which rotated at 10° in every step.

### Supplementary information

Anisotropic phonon dynamics in Dirac semimetal PtTe_2_ thin films enabled by helicity-dependent ultrafast light excitation

## Data Availability

The data that support the plots in this paper and other findings of this study are available from the corresponding authors upon reasonable request.
