# Supplementary material for: Anisotropic phonon dynamics in Dirac semimetal PtTe2 thin films enabled by helicity-dependent ultrafast light excitation
Source: Light Sci Appl. 2024 Aug 1;13:181. doi: 10.1038/s41377-024-01540-z (PMC11294612; doi:10.1038/s41377-024-01540-z)
Supplement: Supplementary file 1 — Anisotropic phonon dynamics in Dirac semimetal PtTe2 thin films enabled by helicity-dependent ultrafast light excitation [file 41377_2024_1540_MOESM1_ESM.docx]

**Supplementary Information for**

**Anisotropic phonon dynamics in** **Dirac semimetal PtTe_2_ thin films enabled by helicity-dependent ultrafast light excitation**

Ziyang Li^1,#^, Yequan Chen^2,#^, Anke Song^2^, Jinzhong Zhang^3^, Rong Zhang^2,4^, Zongzhi Zhang^1*^ and Xuefeng Wang^2*^

^1^Key Laboratory of Micro and Nano Photonic Structures (MOE), School of Information Science and Technology, Fudan University, Shanghai 200433, China

^2^Jiangsu Provincial Key Laboratory of Advanced Photonic and Electronic Materials, State Key Laboratory of Spintronics Devices and Technologies, School of Electronic Science and Engineering, Collaborative Innovation Center of Advanced Microstructures, Nanjing University, Nanjing 210093, China

^3^Department of Physics, School of Physics and Electronic Science, East China Normal University, Shanghai 200241, China

^4^Department of Physics, Xiamen University, Xiamen 361005, China

^#^These authors contributed equally: Ziyang Li, Yequan Chen

Correspondence: Zongzhi Zhang ([zzzhang@fudan.edu.cn](mailto:zzzhang@fudan.edu.cn)) or Xuefeng Wang (xfwang@nju.edu.cn)

**Supplementary Note 1. Coherent acoustic phonon (CAP)**

Figure 2c shows the dynamic relaxation processes on a longer time scale of 0-100 ps and a low-frequency oscillation mode can be recognized, as indicated by the purple arrows. This low-frequency oscillation is considered to be arising from the longitudinal vibration of CAP mode, which is often seen in metals and semiconductors^1^. Owing to the electron-phonon interaction in the region illuminated by the pump beam pulses, the lattice temperature rises instantly and a longitudinal temperature gradient is induced in the sample, which generates a local strain wave (i.e. CAP) because of thermal expansion. The strain wave propagates away along the direction of the temperature gradient (i.e. from the sample surface to the sapphire substrate) at the sound velocity, which periodically modulates the local dielectric constant of the sample and thus the reflectivity^2,3^, leading to the observed periodic oscillation with a rather low frequency. Furthermore, we find that 10 nm PtTe_2_ film exhibits the clear COP signal, but almost no visible CAP oscillations. When the thickness of the sample is less than the penetration depth of the laser, the thermalization caused by the laser will be uniform along the propagation direction of pump pulse so that the weaker longitudinal temperature gradient cannot drive the obvious CAP.

By applying FFT to the curves of Fig. 2c, the central frequency of CAP mode is obtained to be *f*~22.0 GHz (~0.09 meV), as displayed in Fig. 2d. According to the strain wave model, the correlation between *f* and the propagating velocity *υ*_s_ can be expressed as^1,4^

$f=\frac{2n(\lambda)\upsilon_{\text{s}}\cos\theta_{\text{probe}}}{\lambda_{\text{probe}}}$ S(1)

where *θ*_probe_ and *λ*_probe_ denote the incidence angle (~20°) and wavelength (800 nm) of the probe beam, respectively. *n* is the refraction index, which is assumed to be ~4.0 for PtTe_2_ at 800 nm. Based on equation S(1), the sound velocity of PtTe_2_ is calculated to be ~2.3 km s^-1^ at room temperature. Furthermore, according to the relation of *Y* = *ρυ_s_*^2^, the Young’s modulus *Y* is determined as ~51.8 GPa with the density *ρ* = 9.8×10^3^ kg m^-3^ for bulk PtTe_2_^5^, in good agreement with the previous data of PtTe_2_^3^ and other layered TMD materials^6^.

**Supplementary Note 2. Theoretical model of the transient stimulated Raman scattering**

Figure S1 shows the anisotropic phonon dynamics as a function of probe angle *α* via LP excitation. During the impulsive stimulated Raman scattering process in the pump-probe measurements, the relative change of the anisotropic transient reflectivity signal (Δ*R*/*R*) can be written as^7,8^

$(\frac{\Delta R}{R})_{\text{i}=1,2}\propto(e_{\text{pump}}\cdot R\cdot e_{\text{pump}}^{\text{t}})(e_{\text{probe},\text{i}}\cdot R\cdot e_{\text{probe},\text{i}}^{\text{t}})$ S(2)

where i represents the “1” or “2” detection channel of a balanced photodetector, ***e***_pump_ and ***e***_probe_ are the pump and probe electric field unit vector, and ***R*** is the Raman tensor in response to the different phonon modes. The superscript “t” stands for matrix transpose. Here, the pump beam is perpendicularly incident to the film plane, so the electric field of the pump beam lies on the (001) plane of PtTe_2_. The angle of incidence of the probe beam is around 20°. Given the small angle, the probe beam is also assumed to be approximately perpendicular to the sample surface for simplicity of calculation. The electric field vectors of probe and pump beams are described by angle *α* and *β*, namely ***e***_probe_ = (cos*α*, sin*α*, 0) and ***e***_pump_ = (cos*β*, sin*β*, 0).

Considering there is a half-wave plate in front of a Wollaston beam splitter in the probe path, the reflected probe beam is first tuned to a polarization direction of 45° and then split into two components of S-polarization (S-pol) and P- polarization (P-pol). As a result, the electric fields of probe beam measured at “1” and “2” detection channel are ***e***_probe, 1_ = (cos(*α*+π/4), sin(*α*+π/4), 0) and ***e***_probe, 2_ = (cos(*α*-π/4), sin(*α*-π/4), 0), respectively. Then, the differential electrical signal output from the balanced detector is given

$S=\left|(\frac{\Delta R}{R})_{1}-(\frac{\Delta R}{R})_{2}\right|$ S(3)

Theoretically, the second-order Raman tensor ***R*** of non-degenerate *A*_1g_ mode and doubly degenerate *E*_g_ mode in 1*T*-PtTe_2_ can be expressed as^9^

***R***(*A*_1g_):$\left(\begin{matrix} a & 0 & 0 \\ 0 & a & 0 \\ 0 & 0 & b \end{matrix}\right)$, ***R***(*E*_g_)_1_:$\left(\begin{matrix} c & 0 & 0 \\ 0 & -c & d \\ 0 & d & 0 \end{matrix}\right)$ and ***R***(*E*_g_)_2_:$\left(\begin{matrix} 0 & -c & -d \\ -c & 0 & 0 \\ -d & 0 & 0 \end{matrix}\right)$, S(4)

where *a*, *b*, *c* and *d* are the coefficients. Finally, we can obtain *S_A_*_1g_ 0 due to full symmetry, being consistent with the vanished *A*_1g_ mode in this work. For the *E*_g_ mode, we have *S*(*_E_*_g)1_|2*c*^2^sin(2*α*)cos(2*β*)| for Raman tensor ***R***(*E*_g_)_1_ and *S*(*_E_*_g)2_|2*c*^2^cos(2*α*)sin(2*β*)| for Raman tensor ***R***(*E*_g_)_2_, consistent with the observed fourfold symmetry in Fig. 3a and 3b.


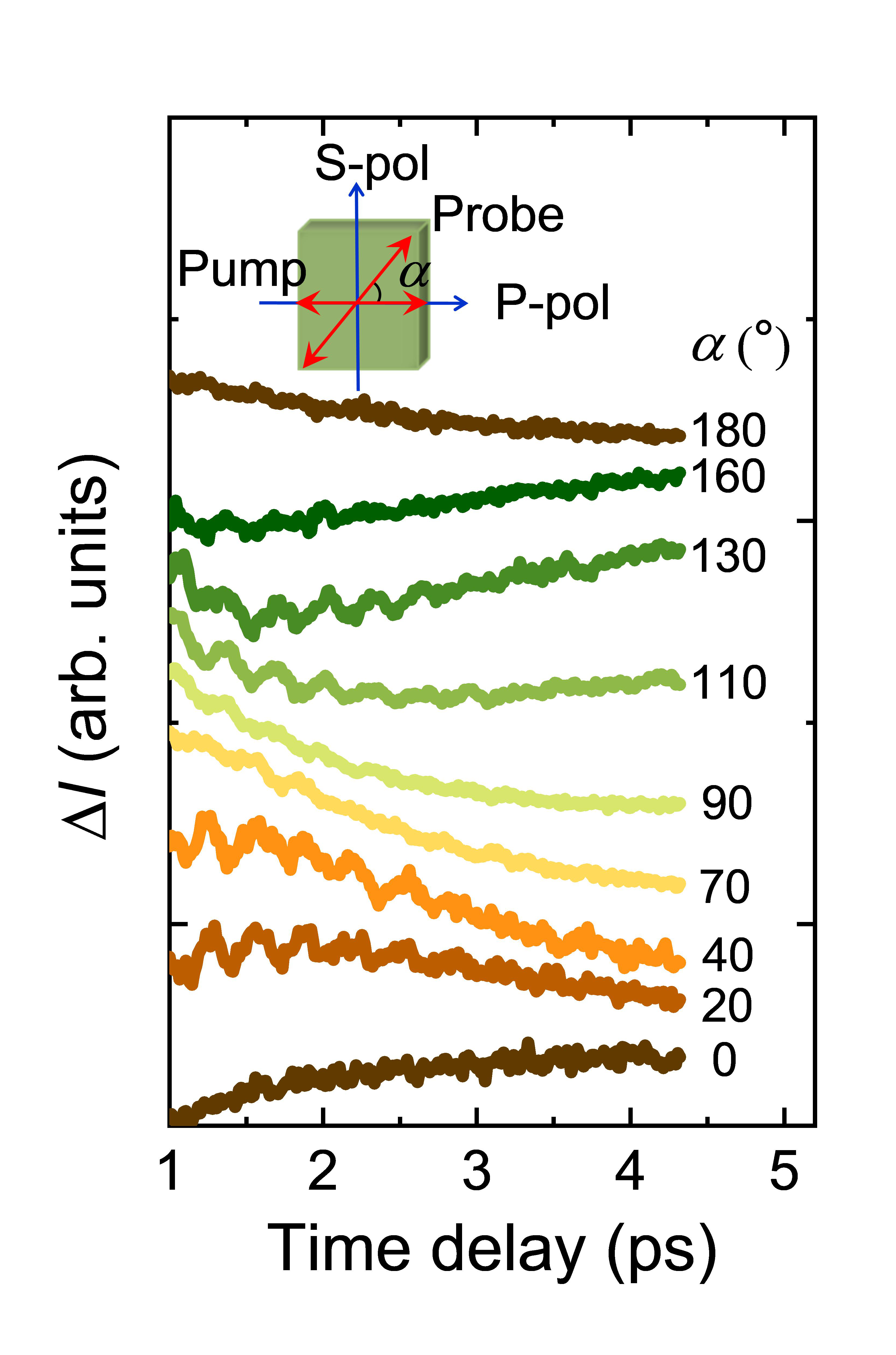


**Fig. S1** **LP excitation.** The time-domain traces of *t* = 1-4.5 ps measured with P-pol pump (*β =* 0) and LP probe beams with polarization angle *α* varying from 0 to 180°. The angle *α* is indicated in the inset.

**Supplementary Note 3. Angle-resolved polarized Raman scattering measurement setup**


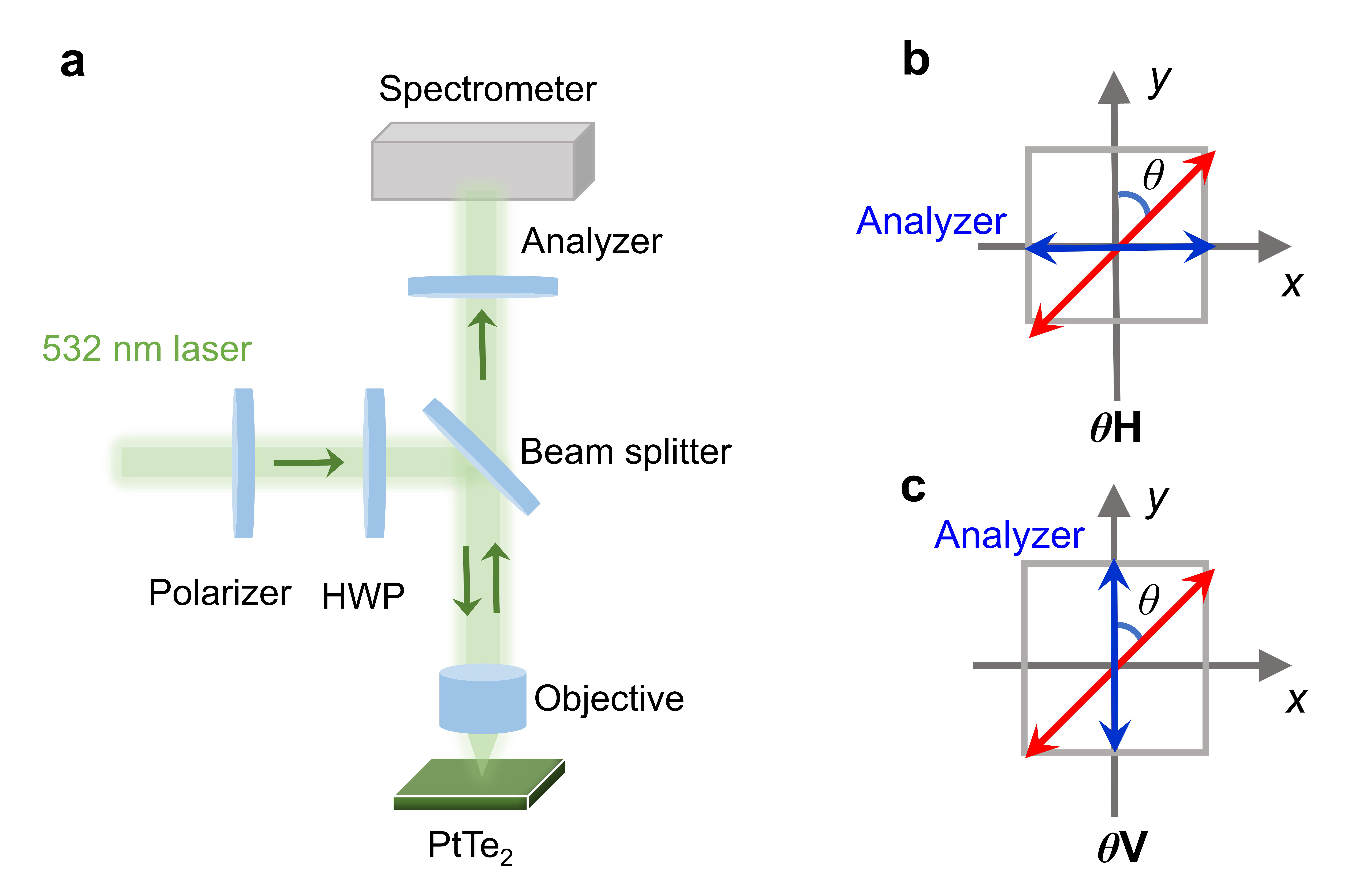


**Fig. S2** **Raman scattering setup.** **a** Schematic diagram of the angle-resolved polarized Raman spectroscopy with 532 nm laser. **b, c** The 𝜃H and 𝜃V configurations of polarized Raman spectroscopy, where the red arrow denotes the incident laser polarization and the blue arrow represents the analyzer polarization.

**Supplementary Note 4. Angle-resolved polarized Raman scattering intensity**

To verify the nonequivalent contributions of two degenerate *E*_g_ modes, angle-resolved polarized Raman spectroscopy was measured by rotating the incident light polarization *θ*. Figure S3a and S3b shows the angle-resolved Raman shift mapping for *θ*H and *θ*V configuration, respectively. Based on the semi-classical analysis, the angle-dependent Raman mode intensities (*S*) can be quantitatively express as^10^

$S\propto\sum\left|e_{\text{s}}\cdot R\cdot e_{\text{i}}^{\text{t}}\right|^{2}$ S(5)

in which ***e***_i_ and ***e***_s_ represent the incident and scattering light electric field unit vector, respectively, and ***R*** is the Raman tensor in response to the different phonon modes. The superscript “t” stands for matrix transpose. The doubly degenerate *E*_g_ mode has two Raman tensors so that its Raman scattering intensity is the summation of the different contributions from ***R***(*E*_g_)_1_ and ***R***(*E*_g_)_2_. Different from the pump-probe approach, we cannot separate the respective contributions of from ***R***(*E*_g_)_1_ and ***R***(*E*_g_)_2_ here. In this measurement, ***e***_i_ can be described with (sin*θ*, cos*θ*, 0) for both configurations, and ***e***_s_ is (1, 0, 0) for *θ*H configuration and (0, 1, 0) for *θ*V configuration. Applying different ***R*** for each Raman mode, we can get the corresponding relation between lattice vibration intensity *S* and polarization direction angle *θ*, as shown below:

1. for the non-degenerate *A*_1g_ mode, $S_{\theta\text{H},A_{1g}}\propto a^{2}\sin^{2}\theta$ and $S_{\theta\text{V},A_{1g}}\propto a^{2}\cos^{2}\theta$;
2. for Raman tensor ***R***(*E*_g_)_1_ in *E*_g_ mode, $S_{\theta\text{H},\text{(}E_{g}\text{)}_{1}}\propto c^{2}\sin^{2}\theta$ and $S_{\theta\text{V},\text{(}E_{g}\text{)}_{1}}\propto c^{2}\cos^{2}\theta$;
3. for Raman tensor ***R***(*E*_g_)_2_ in *E*_g_ mode, $S_{\theta\text{H},\text{(}E_{g}\text{)}_{2}}\propto c^{2}\cos^{2}\theta$ and $S_{\theta\text{V},\text{(}E_{g}\text{)}_{2}}\propto c^{2}\sin^{2}\theta$.

For *A*_1g_ mode, the Raman scattering intensity exhibits a distinct uniaxial anisotropic characteristic, with a maximum to minimum ratio as large as ~20. The minimum values occur at *θ*~0 and 180° for *θ*H configuration in Fig. S3c, corresponding to the maximums of *θ*V configuration in Fig. S3d. These experimental results of *A*_1g_ mode are consistent with the theoretical model.

Further, for *E*_g_ mode, by assuming an identical contribution of Raman tensor ***R***(*E*_g_)_1_ and ***R***(*E*_g_)_2_, the theoretical model can predict a constant Raman scattering intensity in both *θ*H and *θ*V configurations. However, our experimental results are not in strict agreement with the proposed scenario. The shapes of Raman intensity in Fig. S3e and S3f are ellipse instead of circle at the (001) plane, with a maximum to minimum ratio as small as ~1.6, indicating that the contributions of ***R***(*E*_g_)_1_ and ***R***(*E*_g_)_2_ are not equal. Moreover, the directions of the major (or minor) axes of the ellipses in Fig. S3e and S3f strongly support that the intensity of ***R***(*E*_g_)_1_ term is slightly larger than that of ***R***(*E*_g_)_2_, probably due to the anisotropic electronic structure of PtTe_2_^11^.





**Fig. S3** **Polarized Raman scattering. a**, **b** The angle-resolved polarized Raman scattering spectroscopy mapping for *θ*H and *θ*V configurations, respectively. The polarization angle of incident laser varies in the range of 𝜃 = 0-360°. The polar plots of Raman scattering intensity for *A*_1g_ mode (**c**,**d**) and *E*_g_ mode (**e**,**f**) in *θ*H and *θ*V configurations. The red solid curves represent the best fitting lines with a sine function.

**Supplementary Note 5. The local temperature upon laser heating effect**

The accuracy of the temperature control in our temperature-dependent experiments can be within 0.1 K. However, due to the unavoidable laser heating effect, the actual temperature at the laser spot area may often be higher than the set temperature. In order to determine the accurate temperature after laser irradiation, we performed further experimental and theoretical studies for Spontaneous Raman scattering and pump-probe method.

1. **The Stokes and anti-Stokes Raman scattering measurements**

Figure S4a shows the room temperature Raman spectra of PtTe_2_ films with a thickness of ~20 nm. The incident laser has a wavelength of 532 nm and the laser power varies in the range of 0.5-2.0 mW. Apparently, both the *E*_g_ and *A*_1g_ mode are observed. Figure S4b displays the ratio of Stokes and anti-Stokes scattering intensities (*I*_Stokes_ / *I*_anti-Stokes_) as a function of the laser power for both modes. The temperature at the laser spot area can be obtained according to the relation of

$\frac{I_{\text{S}}}{I_{\text{AS}}}=\left(\frac{f_{\text{light}}-f}{f_{\text{light}}+f}\right)^{4}e^{hf/k_{\text{B}}T}$ S(6)

where *f*_light_ and *f* denote the frequency of laser source and the phonon, respectively. As shown in Fig. S4c, when the laser power increases from 0.5 to 2.0 mW, the calculated temperature increase (Δ*T*) of the two modes is ~40 K. The corresponding frequencies of the two mode phonons are shown in Fig. S4d and S4e. For the 2.0 W laser power, the frequency reduction is ~ 0.02 THz, resulting from the ~ 40 K increase in the actual temperature. Since the applied laser power is only 1.0 mW when performing the temperature-dependent Raman scattering measurements (Fig. 5b), the temperature increase induced by the laser heating effect is estimated within ~25 K.

**(2) The two-temperature model calculation in pump-probe experiment**

The temperature variation from the ultrafast laser heating effect can be estimated by using the two-temperature model, which has been widely used in analyzing the transient temperature changes of electron and lattice system after laser excitation. The two-temperature model can be expressed as

$\begin{matrix} C_{\text{e}}\frac{dT_{\text{e}}}{dt}=-G_{\text{el}}(T_{\text{e}}-T_{\text{l}})+P(t) \\ C_{\text{l}}\frac{dT_{\text{l}}}{dt}=G_{\text{el}}(T_{\text{e}}-T_{\text{l}}) \end{matrix}$ S(7)

where *T*_e_ (*T*_l_) denotes the electronic (lattice) temperature, *C*_e_ (*C*_l_) is the electronic (lattice) specific heat capacity, *G*_el_ is the electron-phonon coupling constant. *P* is the laser power density absorbed in the material, which can be modelled by a Gaussian function.

To accurately determine the temperatures of *T*_e_ and *T*_l_, the parameters of *C*_e_, *C*_l_, and *G*_el_ need to be identified. We refer to the experimentally measured heat capacity (*C*) data for single-crystal PtTe_2_^12^. The temperature-dependent *C* is replotted and shown in Fig. S5, where the unit of J mol^-1^ K^-1^ used in ref. 12 is converted to J m^-3^ K^-1^ for the convenience of calculation. As shown in the inset of Fig. S5, the low-temperature data (*T* < 9 K) can be described with *C*/*T* = *γT* + *AT*^2^, which separates the contributions of electrons and phonons. First, the electronic specific heat is expressed as *C*_e_ = *γT*_e_, with a Sommerfeld coefficient of *γ* = 130.84 J m^-3^ K^-2^ obtained by linear fitting. Second, we use the piecewise function to describe the lattice specific heat *C*_l_ in Fig. S5: when *T*_l_ ≥ *θ*_D_ (the Debye temperature *θ*_D_ is ~ 250 K), *C*_l_ is independent of *T*_l_ with the value of *C*_l_ = *C*(300 K) - *C*_e_(300 K) = 1.65×10^6^ J m^-3^ K^-1^, i.e. Dulong-Petit law. When 85 K ≤ *T*_l_ < *θ*_D_, *C*_l_ is temperature-dependent, which is quantitatively depicted by a polynomial fitting with *C*_l_ = (-1.15×10^6^ + 5.64×10^4^×*T* - 5.26×10^2^×*T*^2^ + 2.67×*T*^3^ – 7.11×10^-3^×*T*^4^ + 7.69×10^-6^×*T*^5^) J m^-3^ K^-1^. Last, the electron-phonon coupling constant of *G*_el_ is generally on the order of 10^16^ for semimetals. Here we choose a value of 3.48×10^16^ W m^-3^ K^-1^ for the numerical calculation. A higher or lower magnitude on this order would not affect the calculated lattice temperature in the thermal equilibrium state.

The numerically calculated temporal evolutions of *T*_e_ and *T*_l_ are shown in Fig. S6a for the pump laser fluence of 400 μJ cm^-2^. The electron and lattice system reach thermal equilibrium faster at 85 K (~4.5 ps), as compared to that at 295 K (~7.5 ps). Here, we focus on the lattice temperature of *T*_l_, as it correlates directly with the observed COP behavior. Figure S6b and S6c respectively display the calculated time-domain lattice temperatures of *T*_l_ measured at 300 K and 85 K, which are observed to increase with the rising pump fluences. The lattice temperature increase (Δ*T*) for different pump fluences is summarized in Fig. S6d. Notably, for the pump fluence of 400 μJ cm^-2^ used in our temperature-dependent pump-probe measurements (see Fig. 5a), Δ*T* is found to be less than 25 K at both set temperatures of 85 K and 300 K.

The above experimental and computational results reveal that the local temperature variation (Δ*T*) due to the laser heating effect is less than 25 K in both spontaneous Raman scattering and pump-probe measurements. Therefore, the set temperature can be considered as the actual temperature in our study, which has no significant influence on the analysis of temperature-dependent results.





**Fig. S4 Stokes and anti-Stokes Raman scattering.** **a** Spontaneous Raman spectra including Stokes and anti-Stokes scattering measured at various laser powers. **b** The laser power dependences of the ratios of Stokes and anti-Stokes scattering intensity for both the *E*_g_ and *A*_1g_ mode. **c-e** The calculated temperature increase Δ*T*, the frequency of *E*_g_ and *A*_1g_ modes as a function of laser power, respectively.


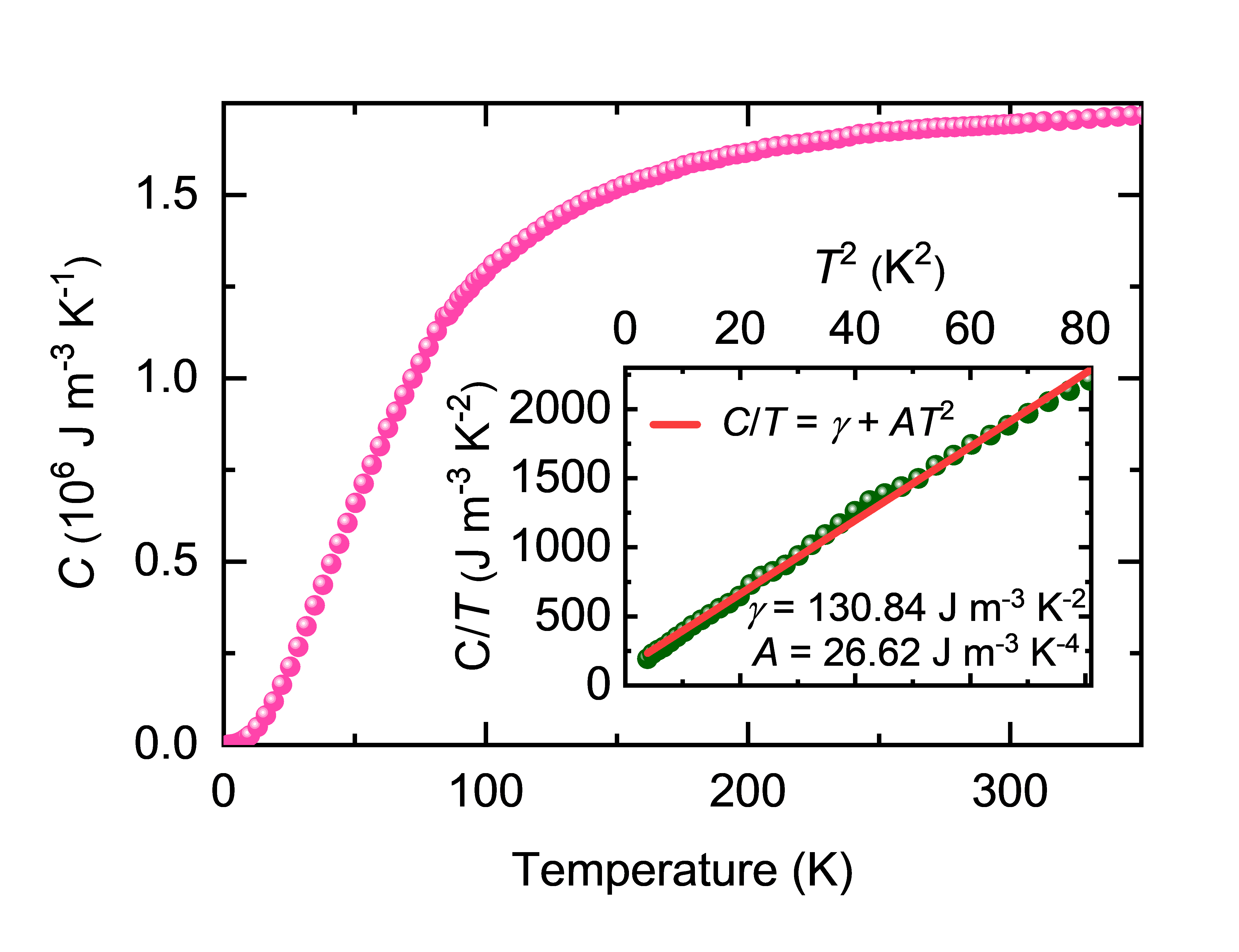


**Fig. S5 Specific heat.** The specific heat of PtTe_2_ below 350 K from the reference^12^ [*Inorg. Mater.*, **59***,* 825–831 (2023)]. The inset shows *C*/*T* versus *T*^2^ at low temperatures.


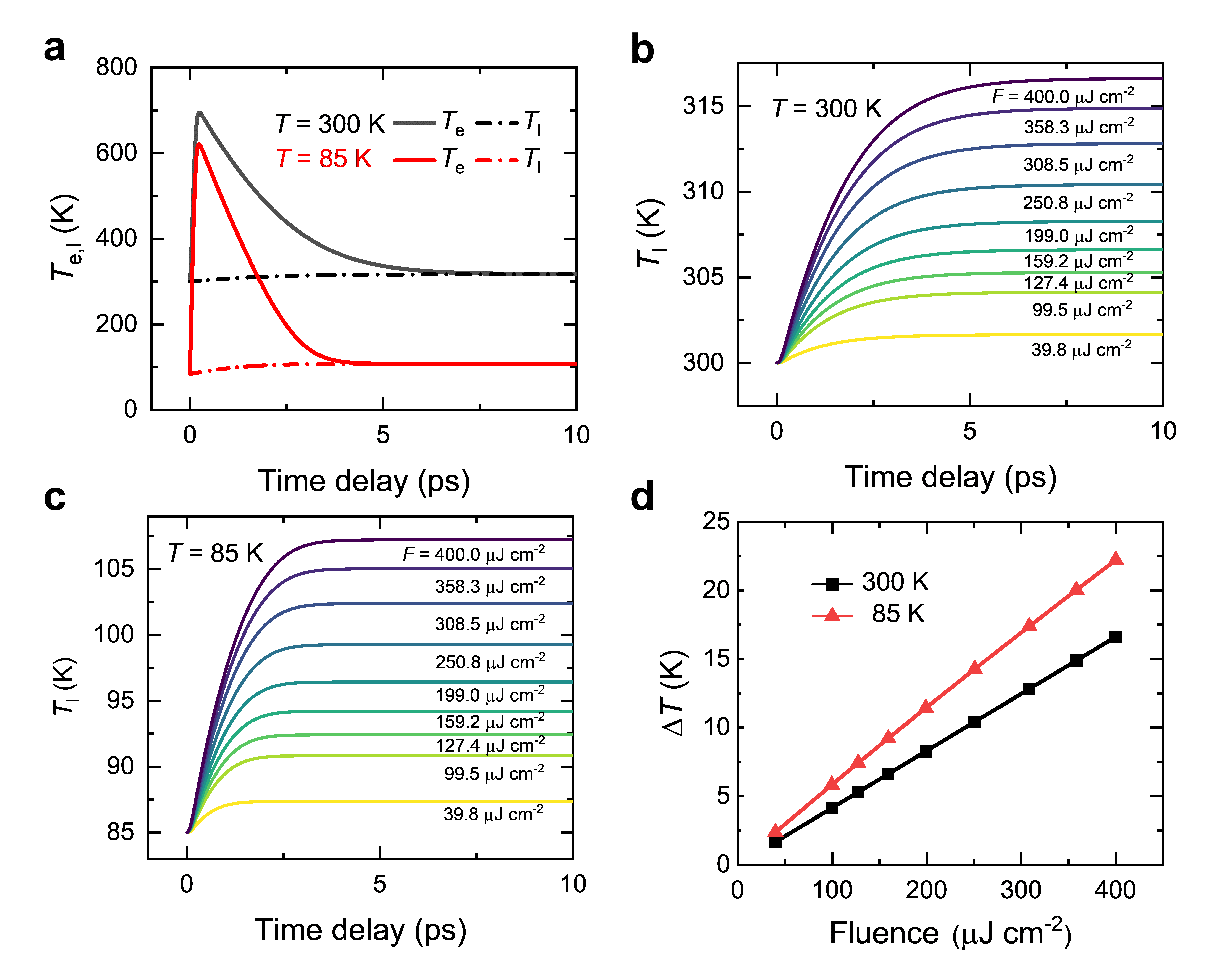


**Fig. S6 Two-temperature model calculation. a** The calculated temporal evolutions of *T*_e_ and *T*_l_ in PtTe_2_ for the pump fluence of 400 μJ cm^-2^ at 85 K and 300 K. **b, c** The temporal evolutions of *T*_l_ for different pump fluence at 85 K and 300 K, respectively. **d** The lattice temperature increase Δ*T* as a function of the pump laser fluence.

**Supplementary Note 6. Pump-fluence-dependent COP dynamics**

The influence of pump laser fluence (*F*) on the lattice dynamics was examined. To avoid damage to the PtTe_2_ sample, the intense pump fluence was varied in the range of 39.8-358.3 μJ cm^-2^. Fig. S7a and S7b show a series of dynamic curves of [Δ*I* (+σ) − Δ*I* (-σ)] measured at *T* = 295 and 85 K, respectively. The fitted *A*_op_ and frequency values are depicted in Fig. S7c and S7d as a function of the pump fluence *F* for *T* = 85 and 295 K, respectively. It is found that *A*_op_ follows a nearly linear dependence, indicating that it is a single photon process, but shows no saturation at even at the highest pump fluence explored. In contrast to *A*_op_, both the frequency curves decrease linearly by ~0.1 THz as the fluence increases from 39.8 to 358.3 μJ cm^-2^. The laser heating effect on temperature increase can be ruled out due to the fact that low pump fluence would not lead to such large temperature changes. These results can be interpreted as an electronic softening of the lattice, associated with the ultrafast photoexcited electron density. At higher pump fluence, more valence band electrons are excited, weakening the restoring force of the *E*_g_-mode lattice displacement^13,14^, and leading to the softening of phonons, as reported in topological semimetals of TiTe_2_^15^. The curves are fitted according to a correlation of *f* = *χ_F_F* + *c*, with nearly identical slopes of *χ_F_*_1_ = -3.37×10^-4^ THz μJ^-1^ cm^2^ for *T* = 85 K and *χ_F_*_2_ = -3.45×10^-4^ THz μJ^-1^ cm^2^ for 295 K, revealing photo absorption of PtTe_2_ are nearly unchanged in the temperature range of 85-295 K. Moreover, the frequency difference value of two fitting lines (Fig. S7d) is ~0.1 THz, perfectly corresponding to the redshift value from 85 K to 295 K in temperature-dependent measurements.





**Fig. S7** **Pump-fluence-dependent COP.** **a**, **b** The time-domain curves of [Δ*I*(+σ) - Δ*I*(-σ)] versus fluence at *T* = 295 K and 85 K, respectively. The solid lines are the fitting curves with equation (1). **c, d** The fitted COP amplitude *A*_op_ and frequency *f* as a function of fluence at *T* = 85 and 295 K, respectively.

**Supplementary Note 7.** **Temperature-dependent frequency and FWHM of the *A*_1g_ mode Raman scattering**

The temperature-dependent frequency and FWHM curves of *A*_1g_ mode in Raman spectra are also analyzed. As shown in Fig. S8a, the frequency value exhibits a distinct redshift with increasing temperature, which originates from phonon anharmonicity including the dominating three-phonon scattering and secondary four-phonon scattering. However, the *A*_1g_ linewidth increases monotonically with increasing temperature in Fig. S8b, which is different from the *E*_g_ mode. The good fitting result reveals that the decay process of *A*_1g_ mode is mainly driven by four-phonon scattering. The electron-phonon coupling contribution of *A*_1g_ mode is much smaller than that of the *E*_g_ mode.


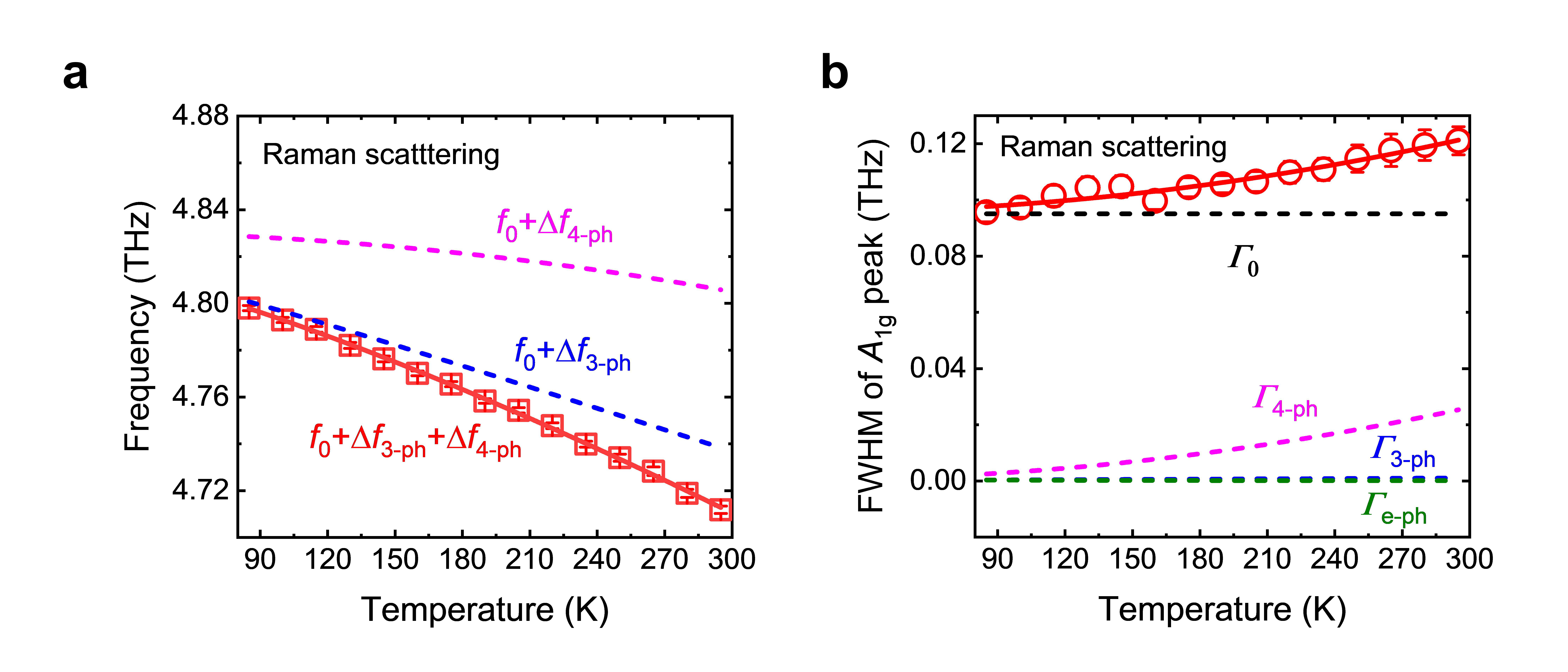


**Fig. S8** **Temperature-dependent *A*_1g_ mode.** **a**, **b** The temperature dependences of the vibration frequency *f* and FWHM of the *A*_1g_ mode in Raman spectra. The red solid lines correspond to the best fitting based on equation (2-4) for (**a**) and equation (5-7) for (**b**). The blue, purple and green dotted lines show the contributions of three-phonon (3-ph), four-phonon (4-ph), and electron-phonon (e-ph) coupling, respectively.

**Supplementary Note 8.** **The fitting parameters of temperature-dependent COP frequency and decay rate**

**Table S1.** The fitting parameters in temperature-dependent pump-probe and Raman scattering measurements.

| Method | Mode | *f*_0_  (THz) | *A*  (GHz) | *B*  (GHz) | *Γ*_0_  (THz) | *C*  (GHz) | *D*  (GHz) | *Γ*_e-ph,0_  (THz) |
| --- | --- | --- | --- | --- | --- | --- | --- | --- |
| Pump-probe | *E*_g_ | 3.32 | -27.5 | 0.477 | 0.71 | 0.37 | 0.164 | 0.76 |
| Raman scattering | *E*_g_ | 3.48 | -19.6 | 0.001 | 0.19 | 0.37 | 0.605 | 0.21 |
|  | *A*_1g_ | 4.83 | -18.0 | -0.566 | 0.10 | 0.20 | 0.590 | 5.71×10^-4^ |

**References**

1 Thomsen, C., Grahn, H. T., Maris, H. J. & Tauc, J. Surface generation and detection of phonons by picosecond light pulses. *Phys. Rev. B* **34**, 4129-4138 (1986).

2 Ge, S. et al. Coherent longitudinal acoustic phonon approaching THz frequency in multilayer Molybdenum Disulphide. *Sci. Rep.* **4**, 5722 (2014).

3 Suo, P. et al. Ultrafast photocarrier and coherent phonon dynamics in type-II Dirac semimetal PtTe_2_ thin films probed by optical spectroscopy. *Photonics Res.* **10**, 653-661 (2022).

4 Cheng, L. et al. Temperature-dependent ultrafast carrier and phonon dynamics of topological insulator Bi_1.5_Sb_0.5_Te_1.8_Se_1.2_. *Appl. Phys. Lett.* **104**, 211906 (2014).

5 Soled, S., Wold, A. & Gorochov, O. Crystal-Growth and Characterization of Platinum Ditelluride. *Mater. Res. Bull.* **10**, 831-835 (1975).

6 Vialla, F. & Del Fatti, N. Time-Domain Investigations of Coherent Phonons in van der Waals Thin Films. *Nanomaterials* **10**, 2543 (2020).

7 Shalini, A. et al. Observation of T_2_-like coherent optical phonons in epitaxial Ge_2_Sb_2_Te_5_/GaSb(001) films. *Sci. Rep.* **3**, 2965 (2013).

8 Norimatsu, K. et al. Dynamics of all the Raman-active coherent phonons in Sb_2_Te_3_ revealed via transient reflectivity. *J. Appl. Phys.* **117**, 143102 (2015).

9 Ribeiro-Soares, J. et al. Group theory analysis of phonons in two-dimensional transition metal dichalcogenides. *Phys. Rev. B* **90**, 115438 (2014).

10 Liu, X. L., Zhang, X., Lin, M. L. & Tan, P. H. Different angle-resolved polarization configurations of Raman spectroscopy: A case on the basal and edge plane of two-dimensional materials. *Chin. Phys. B* **26**, 067802 (2017).

11 Lacinska, E. M. et al. Raman Optical Activity of 1*T*-TaS_2_. *Nano Lett.* **22**, 2835-2842 (2022).

12 Chareev, D. A., Tyurin, A. V., Polotnyanko, N. A. & Chareeva, P. V. Synthesis and Low-Temperature Thermodynamic Functions of Platinum Ditelluride. *Inorg. Mater.* **59**, 825-831 (2024).

13 Hunsche, S., Wienecke, K., Dekorsy, T. & Kurz, H. Impulsive Softening of Coherent Phonons in Tellurium. *Phys. Rev. Lett.* **75**, 1815-1818 (1995).

14 DeCamp, M. F., Reis, D. A., Bucksbaum, P. H. & Merlin, R. Dynamics and coherent control of high-amplitude optical phonons in bismuth. *Phys. Rev. B* **64**, 092301 (2001).

15 Zhu, S.-X. et al. Temperature evolution of quasiparticle dispersion and dynamics in semimetallic 1*T*−TiTe_2_ via high-resolution angle-resolved photoemission spectroscopy and ultrafast optical pump-probe spectroscopy. *Phys. Rev. B* **103**, 115108 (2021).
